# Synthesis of AuPd alloyed nanoparticles via room-temperature electron reduction with argon glow discharge as electron source

**DOI:** 10.1186/1556-276X-9-405

**Published:** 2014-08-20

**Authors:** Manman Yang, Zongyuan Wang, Wei Wang, Chang-jun Liu

**Affiliations:** 1 Collaborative Innovation Center of Chemical Science and Engineering, School of Chemical Engineering and Technology, Tianjin University, Tianjin 300072, China

**Keywords:** Nanoparticles, Metals and alloys, Au, Pd, Electron reduction

## Abstract

Argon glow discharge has been employed as a cheap, environmentally friendly, and convenient electron source for simultaneous reduction of HAuCl_4_ and PdCl_2_ on the anodic aluminum oxide (AAO) substrate. The thermal imaging confirms that the synthesis is operated at room temperature. The reduction is conducted with a short time (30 min) under the pressure of approximately 100 Pa. This room-temperature electron reduction operates in a dry way and requires neither hydrogen nor extra heating nor chemical reducing agent. The analyses using X-ray photoelectron spectroscopy (XPS) confirm all the metallic ions have been reduced. The characterization with X-ray diffraction (XRD) and high-resolution transmission electron microscopy (HRTEM) shows that AuPd alloyed nanoparticles are formed. There also exist some highly dispersed Au and Pd monometallic particles that cannot be detected by XRD and transmission electron microscopy (TEM) because of their small particle sizes. The observed AuPd alloyed nanoparticles are spherical with an average size of 14 nm. No core-shell structure can be observed. The room-temperature electron reduction can be operated in a larger scale. It is an easy way for the synthesis of AuPd alloyed nanoparticles.

## Background

Alloyed AuPd bimetallic nanoparticles have drawn great attention because of their unique properties for optical, electronic, magnetic, and catalytic applications [1-3]. Especially, AuPd alloyed nanoparticles have been widely investigated as catalysts for benzyl oxidation, direct synthesis of hydrogen peroxide from H_2_ and O_2_, and CO oxidation [1,3]. Currently, a variety of approaches have been reported on the preparation of alloyed AuPd nanoparticles, including chemical reduction [3-5], electrochemical reduction [1,6], thermolysis of double metallic salts [2], and sonochemical reduction [7]. Among all these methods, the chemical reduction is mostly applied. It is normally performed using a reducing agent, like NaBH_4_ or H_2_, in the presence of stabilizer or protective molecule for the size and structure control. With the development of green chemistry, the reduction with less chemicals and lower energy consumption has attracted more and more attentions. In this regard, low-temperature bioreduction has been developed [8-11]. For example, Li and his coworkers [11] reported a green synthesis of Ag-Pd alloyed nanoparticles using the aqueous extract of the *Cacumen platycladi* leaves as reducing agent and stabilizing agent [11]. They found that the biomolecules like saccharides, polyphenols, or carbonyl compounds perform as the reducing agent and (NH)C = O groups are responsible for the stabilization of the AgPd alloyed nanoparticles. Recently, reduction using electron beam has been exploited [12]. The reduction by electron beam can be directly performed with electricity only. No chemicals are needed except the precursors of metal ions. It is a green reduction for only reduction process itself is considered. The disadvantage of the electron beam reduction is that the specific equipment and high vacuum operation are required. On the other hand, some cold plasmas like glow discharge, radio frequency (RF) discharge, and microplasma contain a large amount of electrons. These energetic electrons can be employed as the reducing agent. Mougenot et al*.*[13] reported a formation of surface PdAu alloyed nanoparticles on carbon using argon RF plasma reduction. Mariotti and Sankaran [14] and Yan et al*.*[15] reported a microplasma reduction for synthesis of alloyed nanoparticles at atmospheric pressure. These represented a remarkable progress in the green and energy-efficient synthesis of alloyed nanoparticles.

Herein, we report a simple and facile method for the preparation of AuPd alloyed nanoparticles on the anodic aluminum oxide (AAO) surface using room-temperature electron reduction with argon glow discharge as electron source. This reduction operates in a dry way. It requires neither chemical reducing agent nor capping agent. The influence of chemicals on the formed nanoparticles can be eliminated. Glow discharge is well known as a conventional cold plasma phenomenon with energetic electrons. It has been extensively applied for light devices like neon lights and fluorescent lamps. It has also been employed for the preparation of nanoparticles and catalysts [16-20].

## Methods

### Synthesis of AuPd alloyed nanoparticles

**Figure 1:**
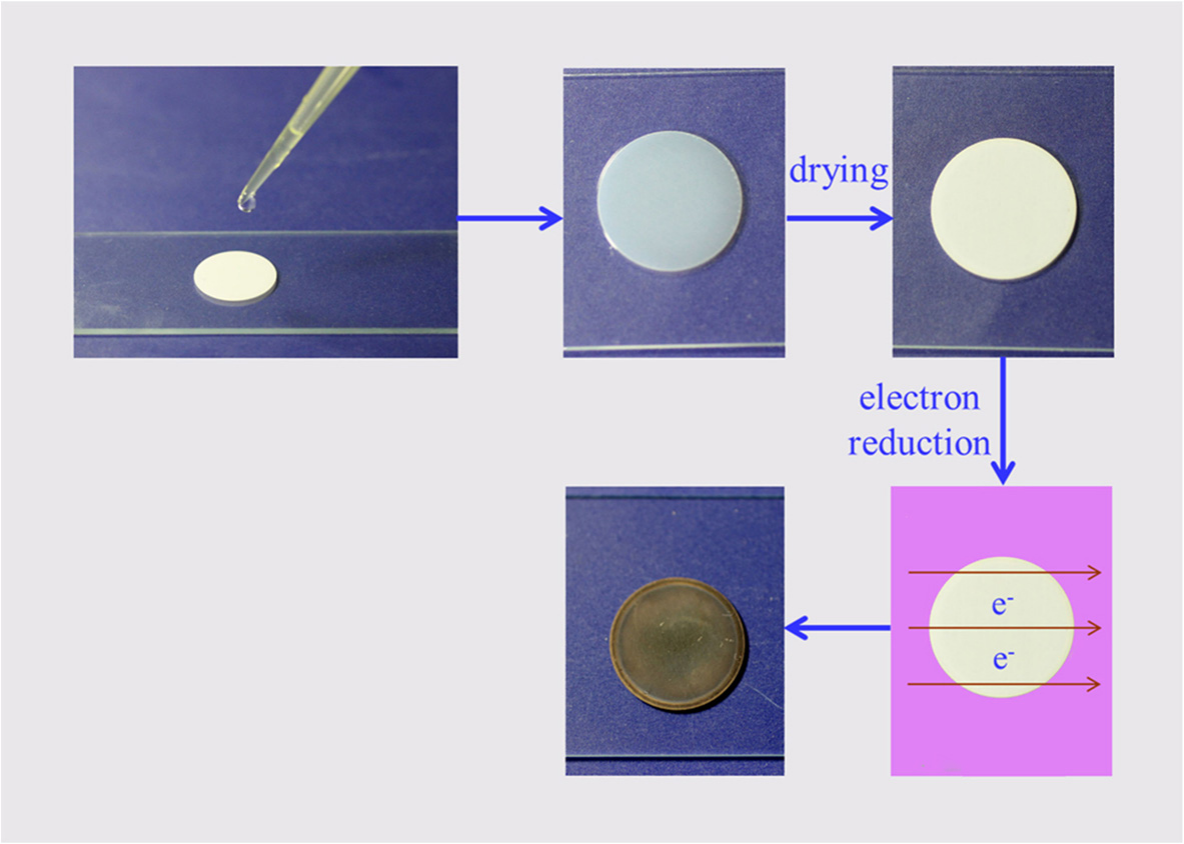
Schematic representative of the electron reduction for the synthesis of AuPd bimetallic nanoparticles.

**Figure 2:**
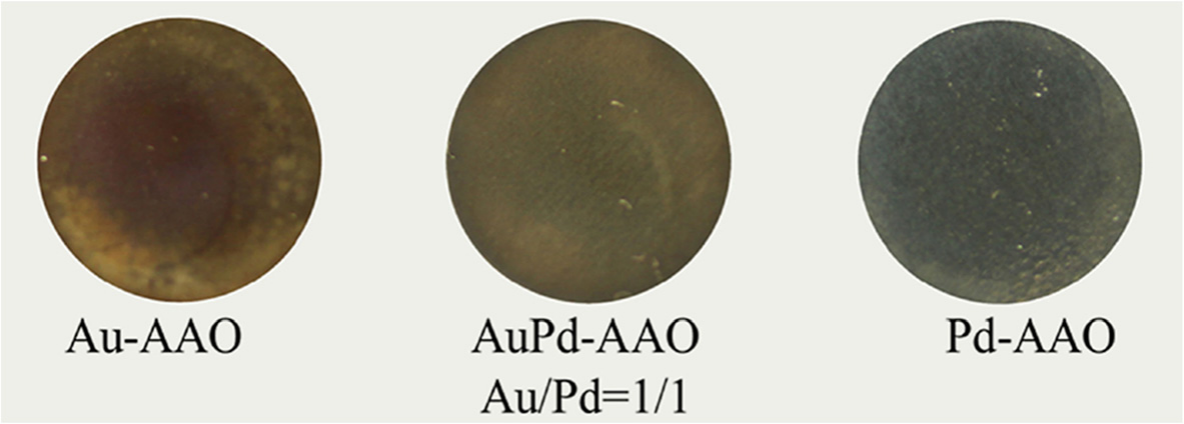
Images of the samples.

AAO with 0.02-μm hole (0.1 mm in thickness, 13 mm in diameter; Whatman International Ltd., Germany) was used as substrate. A solution of HAuCl_4_ and PdCl_2_ was used as metal precursors. A drop of the solution (approximately 30 μL) was dropped on the AAO surface and spread out spontaneously. Then, the AAO sample was put on a glass slide. Once the liquid volatilized, the slide was placed into the glow discharge tube. The pressure of the discharge tube was set at approximately 100 Pa. The argon glow discharge was then initiated by applying high voltage (approximately 1,000 V) using a high-voltage generator (TREK 20/20B, TREK, Inc., Lockport, NY, USA) to the gas. The electron reduction via Ar glow discharge was operated for 3 min. The above steps were repeated for another nine times. Then, bimetallic AuPd nanoparticles were formed. The obtained sample is assigned as AuPd-AAO. Figure 1 shows a schematic representative of the reduction process. The ‘red arrows’ in the figure indicate the direction of electric field. The room-temperature operation was confirmed by thermal imaging [17]. The same method was employed to prepare Au-AAO (0.005 mol/L HAuCl_4_) and Pd-AAO (0.005 mol/L PdCl_2_) for the comparison purpose. Figure 2 presents images of Au-AAO, AuPd-AAO, and Pd-AAO. From the images shown in Figure 2, metallic membranes were directly obtained from the room-temperature electron reduction. However, from the transmission electron microscopy (TEM) images and X-ray diffraction (XRD) analyses, as discussed below, the metallic nanoparticle aggregates were exactly obtained.

### Characterization

The XRD patterns of samples were recorded on a Rigaku D/Max-2500 diffractometer (Rigaku, Shibuya-ku, Japan) (Cu-Kα radiation, *λ* = 0.154056 nm). Diffraction data were collected from 10° to 80° (2*θ*) at a scanning speed of 6°/min. The phase identification was made by comparison with the Joint Committee on Powder Diffraction Standards (JCPDSs). UV–Vis absorption spectra of samples were recorded on a Beckman DU-8B UV–Vis spectrophotometer (Beckman Coulter, Inc., Fullerton, CA, USA). TEM measurements were carried out with a Philips Tecnai G2 F20 system (Philips, Amsterdam, the Netherlands) operated at 200 kV.

## Results and discussion

**Figure 3:**
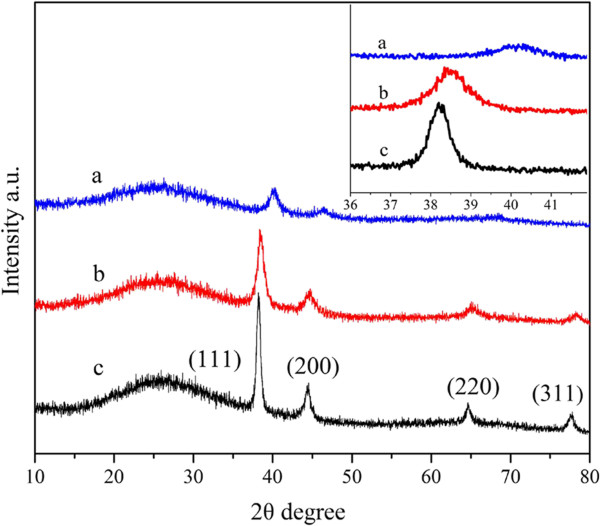
**XRD patterns.** Pd-AAO (a), AuPd-AAO with Au/Pd of 1/1 (b), and Au-AAO (c); enlarged XRD patterns (111 plane) (inset).

**Figure 4:**
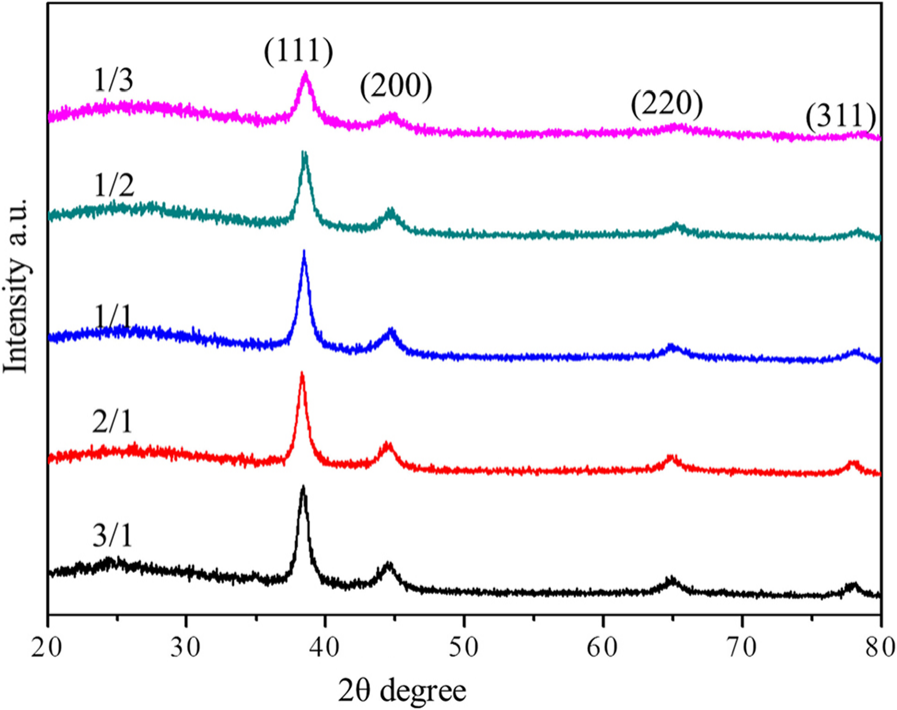
XRD patterns of AuPd-AAO samples with various Au/Pd molar ratios (from 1/3 to 3/1).

The wide-angle XRD patterns of Au-AAO, AuPd-AAO (with Au/Pd molar ratio of 1/1), and Pd-AAO samples are shown in Figure 3. Au-AAO exhibits four diffraction peaks, assigned to (111), (200), (220), and (311) of the face central cubic (fcc) structure of monometallic Au. Pd-AAO presents two diffraction peaks, assigned to (111) and (200) of the fcc structure of monometallic Pd. The bimetallic AuPd-AAO shows four diffraction peaks. However, these four peaks are observed at different 2*θ*, compared to monometallic Au and monometallic Pd samples. The XRD patterns of AuPd-AAO show a big peak at 38.54°, which is between pure Au (111) plane (38.184°; PDF# 04-0784) and pure Pd (111) plane (40.118°; PDF# 46-1043). These results suggest that alloyed bimetallic nanoparticles are formed over AuPd-AAO [4]. According to Vegard's law [2], the Au/Pd molar ratio of the alloyed AuPd sample is approximately 8:2. From XPS analyses, all metal ions have been reduced. However, the peaks belonging to Au and Pd particles cannot be identified from the XRD patterns. This suggests that the formed Au and Pd particles (in addition to alloyed nanoparticles) are highly dispersed and are too small to be observed in the XRD patterns. Similar results were obtained for AuPd-AAO samples with different Au/Pd molar ratios. Only alloyed AuPd nanoparticles can be observed too from the XRD patterns. The size of the alloyed AuPd nanoparticles reduces with the increasing Pd content, as shown in Figure 4.

**Figure 5:**
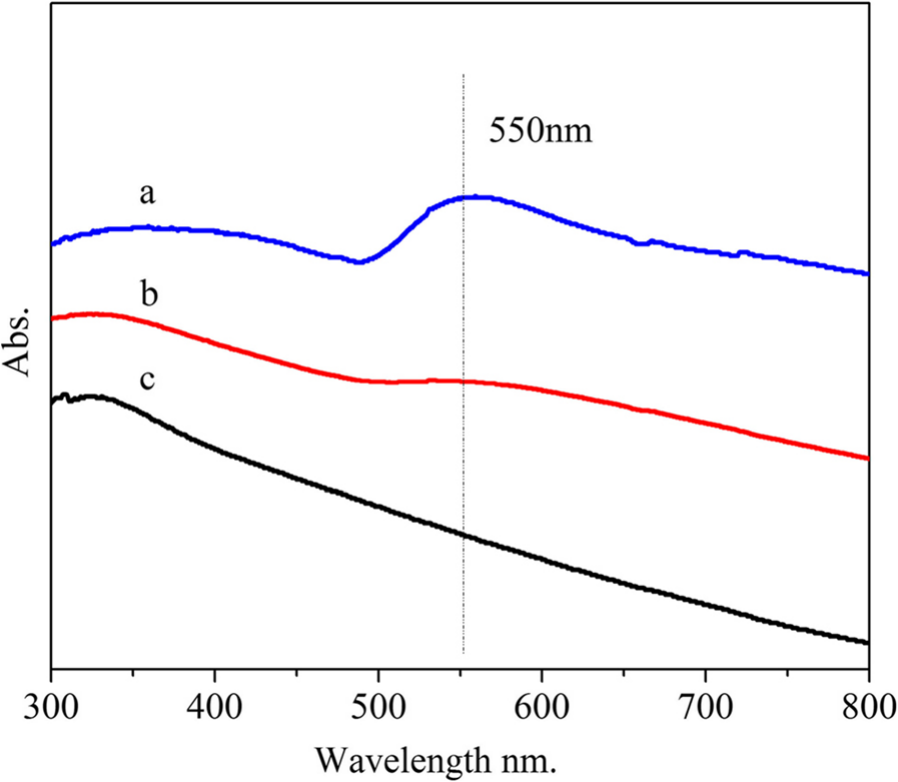
UV–Vis spectra of Au-AAO (a), bimetallic AuPd-AAO with Au/Pd of 1/1 (b), and Pd-AAO (c).

Figure 5 shows UV–Vis spectra of Au-AAO, Pd-AAO, and AuPd-AAO (with Au/Pd molar ratio of 1/1). Before the measurement, the samples were dissolved in NaOH solution and ultrasonically dispersed. Then, the as-prepared solutions were used to absorb UV-visible light. The monometallic Au sample shows a characteristic surface plasmon resonance (SPR) peak centered at 550 nm, which is attributed to Au nanoparticles. The monometallic Pd sample only shows a broad absorption over the entire range. The SPR peak (550 nm) of the Au nanoparticles is obviously damped in the bimetallic AuPd sample. The diminished plasmon band in the bimetallic samples may be attributed to the alloying interaction between Au and Pd [4]. Moreover, the SPR peak of the Au nanoparticles will be completely damped in the completely alloyed AuPd samples [4]. However, the weak SPR peak, assigned to Au nanoparticles, in the UV–Vis spectra can still be observed with the bimetallic sample. These results suggest AuPd-AAO contains AuPd alloyed nanoparticles and monometallic Au nanoparticles. This is well consistent with the XRD results.

**Figure 6:**
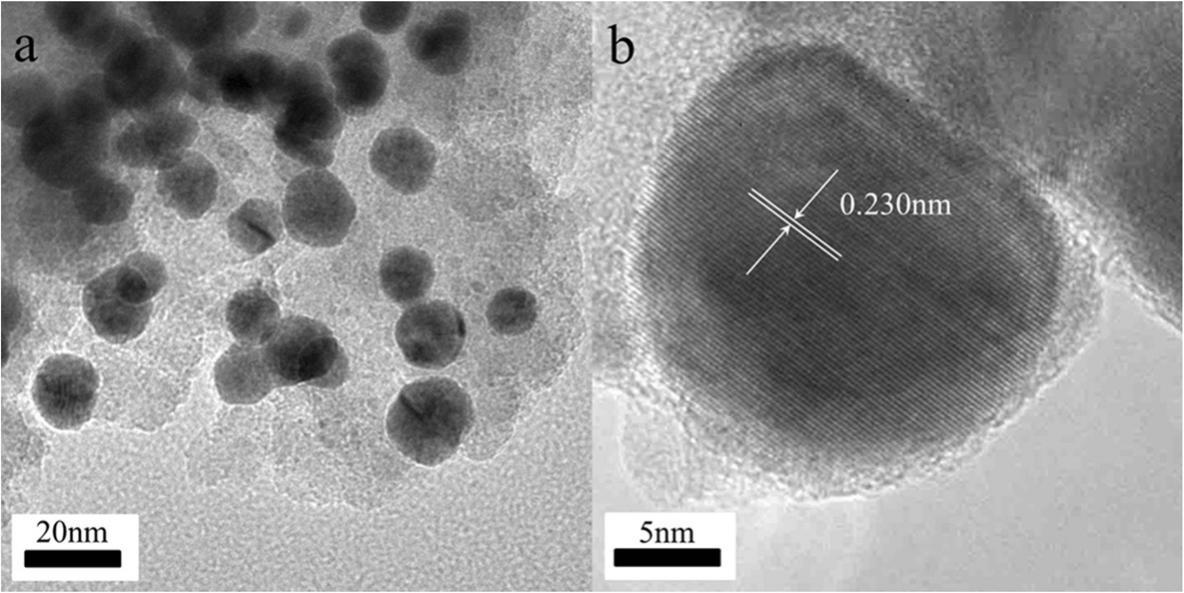
TEM image of AuPd bimetallic nanoparticles with Au/Pd of 1/1 (a) and HRTEM image of AuPd bimetallic nanoparticles (b).

**Figure 7:**
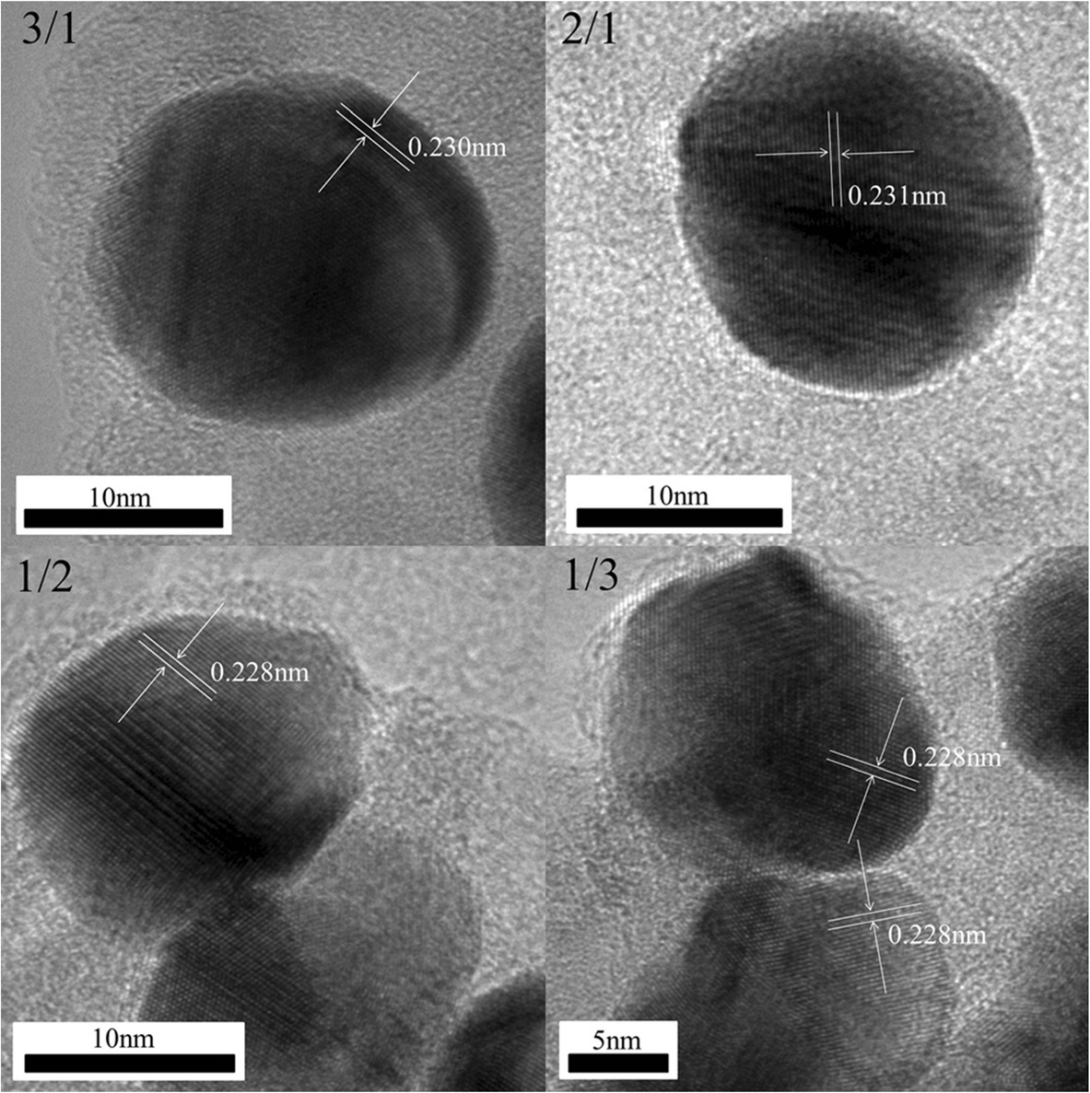
HRTEM images of nanoparticles with different Au/Pd molar ratios.

Figure 6 shows TEM images of AuPd bimetallic nanoparticles (with Au/Pd molar ratio of 1/1). A representative TEM image of AuPd bimetallic nanoparticles is shown in Figure 6a. The AuPd bimetallic nanoparticles are spherical. The average size of the particles is 14 nm. The high-resolution TEM (HRTEM) image of AuPd bimetallic nanoparticle is shown in Figure 6b. No core-shell structure can be observed in the HRTEM image. The d-spacing of the adjacent (111) lattice of the bimetallic nanoparticles is 0.230 nm, while those of the individual Au nanoparticles and Pd nanoparticles are 0.236 and 0.225 nm, respectively. This is well consistent with the (111) plane of AuPd alloyed particles [21-23]. Similar results were obtained for AuPd-AAO samples with different Au/Pd molar ratios, as shown in Figure 7. The d-spacing of the adjacent (111) lattice of bimetallic nanoparticles with different Au/Pd molar ratios is also between those of the individual Au nanoparticles (0.236 nm) and Pd nanoparticles (0.225 nm). Obviously, the TEM analyses confirm the XRD results, and AuPd alloyed nanoparticles are formed with the room-temperature electron reduction.

## Conclusions

In summary, we have successfully prepared AuPd alloyed nanoparticles on the surface of AAO in a dry way directly via room-temperature electron reduction using argon glow discharge as the electron source. The XRD and TEM analyses confirm a formation of AuPd alloyed nanoparticles. The reduction is conducted with a short time (30 min) under the pressure of approximately 100 Pa. The room-temperature electron reduction provides us an easy, direct, green, and cheap way to fabricate AuPd alloyed nanoparticles. This study is leading to further fundamental study of formation of AuPd alloyed nanoparticle.

## Abbreviations

XRD: X-ray diffraction; TEM: transmission electron microscopy; HRTEM: high-resolution transmission electron microscopy.

## Competing interests

The authors declare that they have no competing interests.

## Authors’ contributions

Experiments were designed by CJL and MMY and performed by MMY, ZYW, and WW. Results were analyzed and interpreted by MMY, ZYW, and WW. The manuscript was written by MMY and CJL. CJL is in charge of the project direction, planning, and organization. All authors read and approved the final manuscript.
